# Postoperative collateral formation after indirect bypass for hemorrhagic moyamoya disease

**DOI:** 10.1186/s12883-020-1612-z

**Published:** 2020-01-17

**Authors:** Peicong Ge, Qian Zhang, Xun Ye, Xingju Liu, Xiaofeng Deng, Jia Wang, Rong Wang, Yan Zhang, Dong Zhang, Jizong Zhao

**Affiliations:** 1 Department of Neurosurgery, Beijing Tiantan Hospital, Capital Medical University, Beijing, 100070 China; 2 China National Clinical Research Center for Neurological Diseases, Beijing, China; 3 Center of Stroke, Beijing Institute for Brain Disorders, Beijing, China; 4 Beijing Key Laboratory of Translational Medicine for Cerebrovascular Disease, Beijing, China; 5 Beijing Translational Engineering Center for 3D Printer in Clinical Neuroscience, Beijing, China; 6 Savaid Medical School, University of Chinese Academy of Sciences, Beijing, China

**Keywords:** Postoperative collateral formation, Indirect bypass, Moyamoya disease, Digital subtraction angiography, Hemorrhage

## Abstract

**Background:**

The research on postoperative collateral formation for hemorrhagic moyamoya disease (MMD) evaluated by using digital subtraction angiography (DSA) is limited. Our study objective was to investigate the postoperative collateral formation after indirect bypass for hemorrhagic MMD.

**Methods:**

All consecutive inpatients with hemorrhagic MMD who received indirect bypass at Beijing Tiantan Hospital, Capital Medical University from January 2010 through December 2018 were screened. The site of the hemorrhage was classified as either anterior or posterior. Postoperative collateral formation was evaluated on lateral views using the Matsushima scale. Univariate and multivariate logistic regression analyses were carried out to determine the factors influencing postoperative collateral formation.

**Results:**

Six-four patients (64 hemispheres) were included in this study. After a median 8.5 months DSA follow-up, 14 (21.9%) hemispheres had grade A collateral circulation, 13 (20.3%) had grade B, and 37 (57.8%) had grade C. Twenty-seven (42.2%) hemispheres had good postoperative collateral formation and 37 (57.8%) had poor postoperative collateral formation. The univariate logistic regression analyses showed that age at operation (OR, 0.954; 95% CI, 0.908–1.003; *p* = 0.066), hemorrhagic site (OR, 4.694; 95% CI, 1.582–13.923; *p* = 0.005), and PCA involvement (OR, 3.474; 95% CI, 0.922–13.086; *p* = 0.066) may effect postoperative collateral formation. The multivariate logistic regression analyses showed that only anterior hemorrhage (OR, 5.222; 95% CI, 1.605–16.987; *p* = 0.006) was significantly related to good postoperative collateral formation.

**Conclusion:**

Anterior hemorrhage was significantly related to good postoperative collateral formation after indirect bypass.

## Background

Moyamoya disease (MMD) is a chronic cerebrovascular occlusive disorder, that is characterized by progressive occlusion of the internal carotid arteries or their main branches with compensatory of the basal collateral arterial network (moyamoya vessels) [1, 2]. Intracranial ischemia and hemorrhage are the 2 main manifestations associated with this disease [3].

Although intracranial hemorrhage is less common than ischemic attack, it is the main cause of death in MMD patients [4]. Long-term hemodynamic stress to moyamoya vessels is considered as the main cause of the vascular pathologies resulting in hemorrhage [5]. Although it remains controversial, revascularization surgery has been identified as an effective treatment to decrease hemodynamic stress to these vessels in patients with hemorrhagic MMD [5–7]. In addition, direct bypass could improve cerebral blood flow immediately after successful anastomosis between donor and recipient arteries [4], while indirect bypass takes more time to improve the flow, and the effect of surgical revascularization is based on neovascularization from connective tissue [8].

The research on postoperative collateral formation evaluated by using digital subtraction angiography (DSA) is limited, because DSA not only increases the financial burden on patients, but also is an invasive examination. Nevertheless, it is critically important to know the factors associated with postoperative collateral formation after indirect bypass for hemorrhagic MMD, which may help surgeons optimize the procedure. Here, we performed this retrospective study and tried to determine the factors effectingpostoperative collateral formation.

## Methods

### Patient data

**Fig. 1:**
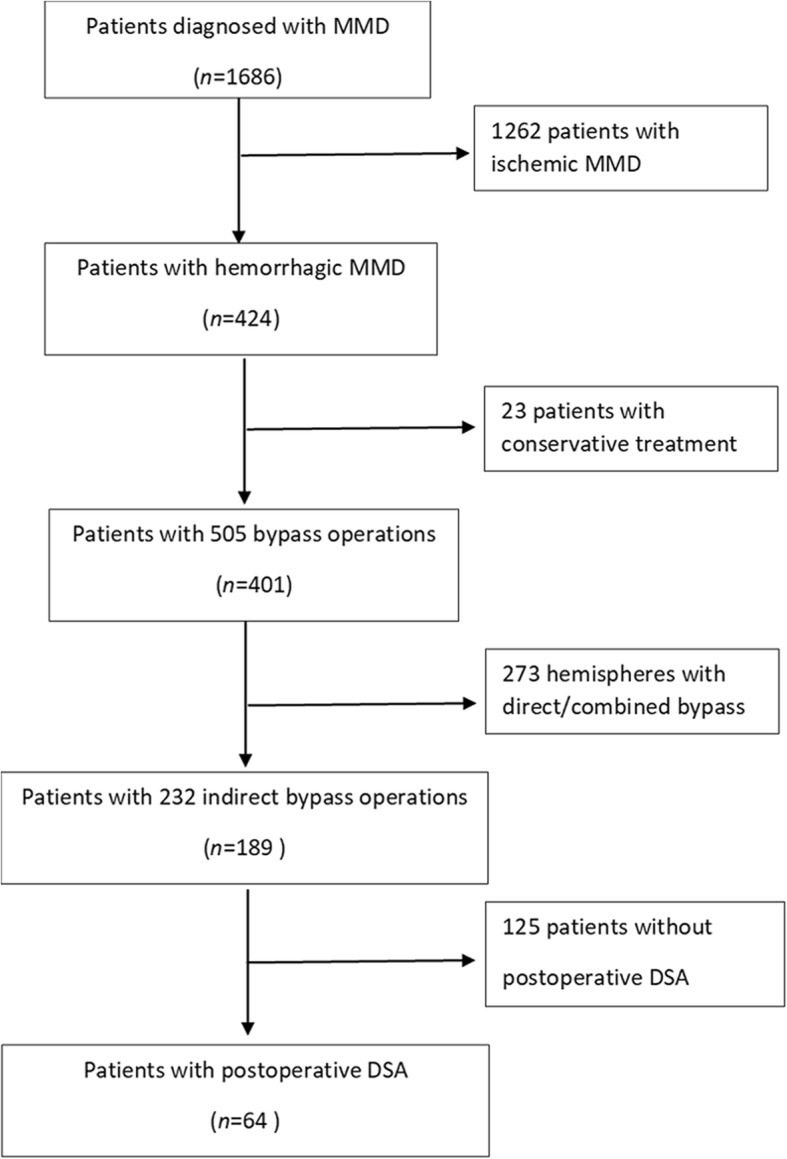
Flow diagram of the study participants

This study was approved by the Ethics Committee of Beijing Tiantan Hospital, Capital Medical University. All consecutive inpatients with MMD at Beijing Tiantan Hospital, Capital Medical University from January 2010 through December 2018 were screened. The inclusion criteria were as follows:1) patients diagnosed with MMD based on DSA according to published guidelines set by the Research Committee on MMD in Japan [9]; 2) patients who initially presented with intracranial hemorrhage confirmed by CT scan; 3) patients who received only indirect bypass; and 4) patients who received postoperative DSA after surgical revascularization. The exclusion criteria included moyamoya syndrome caused by neurofibromatosis, Down syndrome, meningitis, and cranial irradiation [1]. Therefore, 64 patients (64 hemispheres) were included (Fig. 1). Information on the analysis variables, including age at operation, sex, history of risk factors, hypertension, smoking, alcohol use, hyperlipidemia, thyroid disease diabetes, types of hemorrhage, modified Rankin Scale (mRS), and surgical modalities, was collected at study onset.

### Radiologic profiles

The preoperative radiologic profiles, including the site of hemorrhage, collateral circulation,and the stages of the pre-stroke period were determined by two independent neurosurgeons who were blinded to clinical information. The site of hemorrhage was based on the classification criteria established by Takahashi et al. [10]. An anterior hemorrhage is defined as being located in the putamen, caudate head, frontal lobe, anterior half of the temporal lobe, subependymal area of the anterior part of the lateral ventricle, or anterior half of the corpus callosum. A posterior hemorrhage is defined as being located in the thalamus, posterior half of the temporal lobe, parietal lobe, occipital lobe, subependymal area of the posterior part of the lateral ventricle including the atrium, or posterior half of the corpus callosum.

Collateral circulation was evaluated based on the classification criteria by Liu et al. [11]. Posterior collateral circulation was evaluated as follows, based on lateral views of vertebrobasilar artery angiograms, the leptomeningeal collateral networks from the posterior cerebral artery (PCA) territory to the anterior cerebral artery (ACA) territory:1) 1 point: blood supply to the cortical border zone between the ACA and PCA territory; 2) 2 points: blood supply over the central sulcus via the posterior pericallosal artery. On the anteroposterior view vertebrobasilar artery angiograms, the leptomeningeal collateral networks from the PCA territory to the middle cerebral artery (MCA) territory: 1) 1 point: the anastomoses of the anterior temporal branches of the PCA and MCA or the parietooccipital PCA anastomoses to MCA; 2) points: blood supply extended into the sylvian fissure; 3) 3 points: blood supply extended into the occlusion within the M1 or proximal M2 segments. Anterior collateral circulation was evaluated by using the Suzuki stage [12], and scores of 6 to 0 corresponded to Suzuki stages 0 to 6. The grading score was obtained based on the sum of the anterior and posterior collateral circulation and the stages of collateral circulation were made as follows: Grade I, a score of 0 to 4; Grade II, a score of 5 to 8; and Grade III, a score of 9 to12.

The cerebral hemodynamic status was assessed by computed tomography perfusion. The stages of pre-stroke period were evaluated as follows [13]: Stage I, time to peak (TTP) was delayed, mean transit time (MTT), regional cerebral blood flow (rCBF), and regional cerebral blood volume (rCBV) were normal; Stage II, TTP and MTT were delayed, rCBF was normal, and rCBV was normal or slightly increased; Stage III, TTP and MTT were delayed, rCBF was decreased, and rCBV was normal or slightly decreased; Stage IV, TTP and MTT were delayed, rCBF and rCBV were decreased.

### Postoperative collateral formation

**Fig. 2:**
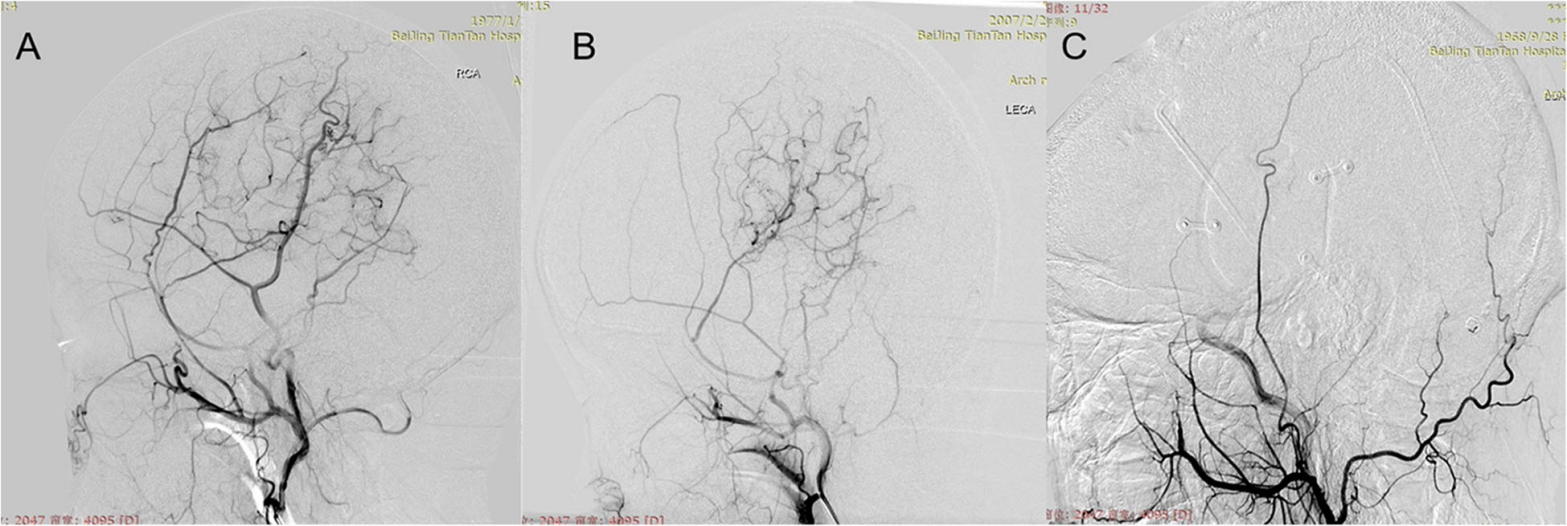
Postoperative collateral formation was evaluated with the Matsushima scale: A, more than 2/3 of the MCA distribution; B, between 2/3 and 1/3 of the MCA distribution. C, slight or none

Direct or combined bypass is the first choice for the treatment of hemorrhagic MMD in our centre. However, direct bypass is difficult in young pediatric patients or adult patients with advanced MMD due to the small caliber of the recipient artery. Indirect bypass was performed unless there were inadequate recipient or donor artery grafts [14], and encephaloduroarteriosynangiosis (EDAS) was the prioritized technique. For patients with no available donor vessels, multiple burr hole (MBH) or encephalodurogaleo (periosteal) synangiosis (EDGS) was performed [15]. For EDAS, the branch of the superficial temporal artery (STA) and the surrounding galea connective tissue were placed on the brain surface after being dissected free, and EDGS was performed as a variant of EDAS. For MBH, five to fifteen burr holes were drilled over the hypoperfusion brain area; the dura was opened and separated. Postoperative collateral formation was evaluated by using the Matsushima scale on lateral views of external carotid angiograms [16]: A, more than 2/3 of the MCA distribution; B, between 2/3 and 1/3 of the MCA distribution; and C, slight or none (Fig. 2). The evaluations were carried out by two independent neurosurgeons who were not involved in the surgical procedures and who were blinded to the clinical information.

### Statistical analysis

The statistical analyses were performed using SPSS (Windows version 22.0, IBM). AnA or B score on the Matsushima scale was defined as good postoperative collateral formation, and a C score on the Matsushima was defined as poor postoperative collateral formation. A logistic regression analysis was performed to test which variables were associated with postoperative collateral formation. Clinical variables that achieved *p* < 0.10 in the univariate analysis were included in the multivariate analysis. A probability value < 0.05 was defined as statistical significance.

## Results

### Baseline characteristics

A total of 64 patients (64 hemispheres) with hemorrhagic MMD who received indirect bypass were enrolled in the study. The mean ± SD age at operation was 36.2 ± 10.7 years (range 9–61 years), and there were 42 female and 22 male patients (female/male ratio was 1.91:1.00). Of the 64 patients, the most common history of risk factor was hypertension (21.9%) And the most common type of hemorrhage on CT was IVH (*n* = 33, 52.6%), followed by ICH with IVH (*n* = 13, 20.3%), ICH (*n* = 13, 20.3%), and SAH (*n* = 3, 4.7%). Most patients received EDAS (*n* = 53, 82.8%), five (7.8%) patients received EDGS, and 6 (9.4%) patients underwent MBH (Table 1).

**Table 1:** Baseline characteristics of 64 patients

Characteristics	Value
Sex ratio (F/M)	42:22
Age at operation, mean ± SD, y	36.2 ± 10.7
Age	
<18 years	5 (7.8%)
History of risk factors	
Hypertension	14 (21.9%)
Smoking	4 (6.3%)
Alcohol use	3 (4.7%)
Thyroid disease	3 (4.7%)
Hyperlipidemia	2 (3.1%)
Diabetes	1 (1.6%)
Type of hemorrhage	
IVH	33 (52.6%)
ICH&IVH	15 (23.4%)
ICH	13 (20.3%)
SAH	3 (4.7%)
mRS > 2 at admission	30 (46.9%)
Surgical modalities	
EDAS	53 (82.8%)
EDGS	5 (7.8%)
MBH	6 (9.4%)
DSA follow-up, median (IQR), mons	8.5 (6–13)

Abbreviations: *DSA* Digital subtraction angiography, *EDAS* Encephaloduroarteriosynangiosis, *EDGS* Encephalodurogaleo (periosteal)synangiosis, *ICH* Intracranial hemorrhage, *IVH* Intraventricular hemorrhage, *MBH* Multiple burr hole, *mRS* Modified Rankin Scale, *SAH* Subarachnoid hemorrhage

### Radiologic profiles

Of the 64 hemispheres with hemorrhagic MMD, 34 hemispheres (53.1%) suffered anterior hemorrhage, and 30 hemispheres (46.9%) suffered posterior hemorrhage (Table 2). Mosthemispheres presented with Suzuki stage III or IV (73.4%), and 12 hemispheres (18.8%) had posterior cerebral artery involvement. Among the 64 hemorrhagic hemispheres, 13 (20.3%) were grade I hemispheres, 48 (75.0%) were grade II hemispheres, and 3 (4.7%) were grade III hemispheres. Angiographic dilation and extension of AChA-PCoA was detected in 48 hemispheres (75%). Superficial temporal artery collateral was found in one (1.6%) hemisphere, middle meningeal artery collateral was detected in 36 (56.3%) hemispheres, and occipital artery collateral was found in 6 (9.4%) hemispheres. The distribution of the stage of the pre-stroke period was as follows, normal, *n* = 9 (14.1%), stage I, *n* = 1 (1.6%); stage II, *n* = 18 (28.1%); stage III, *n* = 18 (28.1%); stage IV, *n* = 18(8.1%).

**Table 2:** Radiologic profiles

Characteristics	Value (%)
Hemorrhagic site	
Anterior	34 (53.1)
Posterior	30 (46.9)
Suzuki stage	
II	7 (10.9)
III	28 (43.8)
IV	19 (29.7)
V	1 (1.6)
VI	2 (3.1)
PCA involvement	12 (18.8)
Collateral circulation	
Grade I (1–4)	13 (20.3)
Grade II (5–8)	48 (75.0)
Grade III (9–12)	3 (4.7)
Dilation of AChA-PCoA	48 (75.0)
ECA collateral	
STA collateral	1 (1.6)
MMA collateral	36 (56.3)
OA collateral	6 (9.4)
The stage of pre-stroke period	
Normal	9 (14.1)
Stage I	1 (1.6)
Stage II	18 (28.1)
Stage III	18 (28.1)
Stage IV	18 (28.1)

Abbreviations: *AChA* Anterior choroidal artery, *ECA* External carotid artery, *MMA* Middle meningeal artery, *OA* Ooccipital artery, *PCA* Posterior cerebral artery, *PCF* Postoperative collateral formation, *PCoA* Posterior communicating artery, *STA* Superficial temporal artery

### Predictors for postoperative collateral formation after indirect bypass

After a median 8.5 month follow-up with DSA, among the 64 hemispheres received indirect bypass, 14 (21.9%) hemispheres had grade A collateral circulation, 13 (20.3%) -had grade B collateral circulation, and 37 (57.8%) had grade C collateral circulation. Twenty-seven (42.2%) hemispheres had good postoperative collateral formation and 37 (57.8%) had poor postoperative collateral formation (Table 3). The univariate logistic regression analysis showed that age at operation (OR, 0.954; 95% CI, 0.908–1.003; *p* = 0.066), hemorrhagic site (OR, 4.694; 95% CI, 1.582–13.923; *p* = 0.005), and PCA involvement (OR, 3.474; 95% CI, 0.922–13.086; *p* = 0.066) may effect postoperative collateral formation. Multivariate logistic regression analysis showed that only anterior hemorrhage (OR, 5.222; 95% CI, 1.605–16.987; *p* = 0.006) was significantly related to good postoperative collateral formation.

**Table 3:** Logistic regression analysis of predictors for postoperative collateral formation

Characteristics	PCF		*p* value		OR (95% CI)
	Good (*n* = 27)	Poor *(n* = 37)	Uni	Multi ^a^	
Age, years	33.3 ± 13.0	38.6 ± 8.3	0.066	0.067	0.948 (0.896–1.004)
Male sex	8 (29.6%)	14 (37.8%)	0.496		
History of risk factors					
Hypertension	7 (25.9%)	7 (18.9%)	0.504		
Smoking	1 (3.7%)	3 (8.1%)	0.483		
Diabetes	1 (3.7%)	0 (0.0%)	1.000		
Alcohol use	0 (0.0%)	3 (8.1%)	0.999		
Hyperlipidemia	1 (3.7%)	1 (2.7%)	0.821		
Thyroid disease	1 (3.7%)	2 (5.4%)	0.752		
Type of hemorrhage					
IVH	14 (51.9%)	19 (51.4%)	0.968		
ICH&IVH	5 (18.5%)	10 (27.0%)	0.430		
ICH	5 (18.5%)	8 (21.6%)	0.824		
SAH	3 (11.1%)	0 (0.0%)	0.999		
Hemorrhagic site			0.005	0.006	5.222 (1.605–16.987)
Anterior	20 (74.1%)	14 (37.8%)			
Posterior	7 (25.9%)	23 (63.2%)			
Suzuki stage			0.823		
II	4 (14.8%)	3 (8.1%)			
III	12 (44.4%)	21 (56.8%)			
IV	9 (33.3%)	12 (32.4%)			
V	1 (3.7%)	0 (0.0%)			
VI	1 (3.7%)	1 (2.7%)			
PCA involvement	8 (29.6%)	4 (10.8%)	0.066	0.067	4.181 (0.906–19.306)
Collateral circulation			0.907		
Grade I (1–4)	6 (22.2%)	7 (18.9%)			
Grade II (5–8)	19 (70.4%)	29 (78.4%)			
Grade III (9–12)	2 (7.4%)	1 (2.7%)			
Dilation of AChA-PCoA	18 (66.7%)	30 (81.1%)	0.193		
ECA collateral					
STA collateral	0 (0.0%)	1 (2.7%)	1.000		
MMA collateral	17 (63.0%)	19 (51.4%)	0.356		
OA collateral	3 (11.1%)	3 (8.1%)	0.685		
The stage of pre-stroke period			0.590		
Normal	3 (11.1%)	6 (16.2%)			
Stage I	0 (0.0%)	1 (2.7%)			
Stage II	9 (33.3%)	9 (24.3%)			
Stage III	12 (44.4%)	6 (16.2%)			
Stage IV	3 (11.1%)	15 (40.5%)			
EDAS surgery	23 (85.2%)	30 (81.1%)	0.668	0.998	0.998 (0.210–4.742)

^a^Adjusted for surgical modalities

Abbreviations: *AChA* Anterior choroidal artery, *ECA* External carotid artery, *EDAS* Encephaloduroarteriosynangiosis, *CI* Confidence intervals, *MMA* Middle meningeal artery, *ICH* Intracranial hemorrhage,*IVH* Intraventricular hemorrhage, *OA* Occipital artery, *OR* Odds ratios, *PCA* Posterior cerebral artery, *PCF* Postoperative collateral formation, *PCoA* Posterior communicating artery, *SAH* Subarachnoid hemorrhage, *STA* Superficial temporal artery

## Discussion

Hemorrhagic MMD was less common than ischemic MMD, but patients with hemorrhagic MMD had higher morbidity, higher mortality rates and worse prognosis than patients with ischemic MMD [17, 18]. Although it remains controversial, revascularization surgery has been identified as an effective treatment for hemorrhagic MMD [19]. The effect of indirect revascularization to improve cerebral blood flow was based on postoperative collateral formation from the ingrowth of new vessels [4, 6, 8]. However, because of the rarity of the disease and the invasiveness of DSA examinations, few studies have focused on postoperative collateral formation after indirect bypass in hemorrhagic MMD patients. In this study, we investigated the relationship between various factors and postoperative collateral formation, and found that anterior hemorrhage was associated with good postoperative collateral formation.

At present, surgical revascularization is considered to improve cerebral blood flow and decrease the rate of stroke events, whereas the optimal treatment for patients with hemorrhagic MMD remains controversial [6, 8]. In Japan, the results of the JAM trial conducted by 22 institutes in Japan showed that direct bypass can decrease the incidence of hemorrhagic events, but the difference was marginally significant [5]. In Korea, Jang et al. showed that bypass surgery reduced stroke recurrence in patients with hemorrhagic MMD [20]. In China, our previous study also showed that surgical revascularization improved cerebral blood flow and had greater efficacy in preventing rebleeding than conservative therapy [21]. Jiang et al. showed that combined bypass may be superior to conservative treatment for patients with hemorrhagic MMD [22]. However, the results of some studies were less optimistic, Ikezaki et al. conducted a nationwide survey of 232 patients, which revealed that there was no significant difference in the rebleeding rate between surgical and conservative treatments [23]. Houkin et al. also showed that revascularization surgery cannot always prevent rebleeding [24]. Although there is still no clear evidence that surgical revascularization significantly prevents rebleeding in adult MMD patients, revascularization surgery is still considered the first choice for the treatment of patients with hemorrhagic MMD in our centre.

There have been a few studies of indirect bypass for patients with hemorrhagic MMD [25–27]. Wang et al. conducted a study of 95 adult hemorrhagic patients after EDAS, and found that EDAS was beneficial for patients with hemorrhagic MMD [25]. And An et al. assessed 13 children with hemorrhagic MMD who received indirect bypass, and revealed that revascularization surgery may have had a role preventing rebleeding [27]. However, Aoki reported that indirect bypass failed to prevent recurrent hemorrhage in patients with hemorrhagic MMD [28]. However our prospective cohort study showed that indirect bypass was similarly effective at preventing recurrent hemorrhagic strokes, compared with combined bypass and direct bypass [14]. A network meta-analysis of hemorrhagic MMD revealed that indirect bypass had a role in treating hemorrhagic MMD [19].

In this study, we investigated postoperative collateral formation. Twenty-seven (42.2%) hemispheres had good postoperative collateral formation and 37 (57.8%) had poor postoperative collateral formation. Good collateral formation was relatively low. However, a recent study showed that 75% of hemispheres were classified as having grade A collateral circulation [25], which was much higher than our study; these two studies had diametrically opposite results, and further research is needed. Takahashi et al. investigated the significance of the hemorrhagic site for recurrent bleeding in the JAM trial [10],and found that patients with posterior hemorrhage had a higher incidence of rebleeding and got greater benefit from direct bypass. Moreover, the results of a study of 95 adult patients with hemorrhagic MMD showed that, after EDAS surgery, the incidence rate was higher for patients with posterior hemorrhage than for patients with anterior hemorrhage, but there was no significant difference [25]. The results of our study revealed that anterior hemorrhage was associated with good postoperative collateral formation, and posterior hemorrhage was related to poor postoperative collateral formation, which may explain why patients with posterior hemorrhage had a higher rate of rebleeding than patients with anterior hemorrhage. The anterior hemorrhage may originate in the lenticulostriate arteries, and posterior hemorrhage may come from the thalamic or choroidal arteries. The lenticulostriate arteries might suffer less hemodynamic stress than the choroidal or thalamic arteries as long as the terminal portion of the internal carotid artery has narrowed [10]. We speculated that hemodynamic stress may influence the ingrowth of new vessels in indirect bypass, and that patients with less hemodynamic stress might have better postoperative collateral formation. These points of speculation should be confirmed through further analysis.

Our study had a few limitations. First, our study was a nonrandomized retrospective, single centre study, so selection bias may exist. Second, the sample size was not larger enough, and there wereonly 64 hemispheres included in this study. Third, the median follow-up with DSA was only 8.5 months, and we could not investigate long-term postoperative collateral formation. However, the general effect of indirect revascularization was very similar in the short and long term follow-up [29]. In the future, long-term clinical follow-up assessments for patients enrolled in this study will be performed, and the rebleeding rate in patients with different hemorrhagic sites and postoperative collateral formation will be evaluated.

## Conclusion

Anterior hemorrhage was associated with good postoperative collateral formation. In the future, we will perform long-term clinical follow-up assessments for patients enrolled in this study, and evaluate the rebleeding rate in patients with different hemorrhagic sites and postoperative collateral formation.

## Data Availability

The datasets supporting the conclusions of this study are available from the corresponding author on reasonable request.

## Abbreviations

AChA: Anterior choroidal artery
DSA: Digital subtraction angiography
ECA: External carotid artery
EDAS: Encephaloduroarteriosynangiosis
EDGS: Encephalodurogaleo (periosteal)synangiosis
ICH: Intracranial hemorrhage
IVH: Intraventricular hemorrhage
JAM: Japan adult moyamoya
MBH: Multiple burr hole
MMA: Middle meningeal artery
mRS: Modified rankin scale
OA: Occipital artery
PCA: Posterior cerebral artery
PCF: Postoperative collateral formation
PCoA: Posterior communicating artery
SAH: Subarachnoid hemorrhage
STA: Superficial temporal artery
